# NCAPG promotes the progression of lung adenocarcinoma via the TGF-β signaling pathway

**DOI:** 10.1186/s12935-021-02138-w

**Published:** 2021-08-21

**Authors:** Yun Wu, Ying Lin, Junfan Pan, Xunwei Tu, Yiquan Xu, Hongru Li, Yusheng Chen

**Affiliations:** 1 Shengli Clinical Medical College of Fujian Medical University, Fuzhou, 350001 China; 2 Department of Pulmonary and Critical Care Medicine, Fujian Provincial Hospital, Fuzhou, 350001 China; 3 Fujian Provincial Researching Laboratory of Respiratory Diseases, Fuzhou, 350001 China

**Keywords:** Lung adenocarcinoma, NCAPG, TGF-β signaling pathway, Prognostic biomarkers

## Abstract

**Background:**

Lung cancer has the highest case fatality rate among cancers because of uncontrolled proliferation and early metastasis of cancer cells in the lung tissue. This study aimed to clarify the role of the non-SMC condensin I complex, subunit G (NCAPG) in lung adenocarcinoma (LUAD), explore the mechanisms of its progression, and lay the foundation for the search for new biological markers.

**Methods:**

We analyzed overlapping differentially expressed genes (DEGs) from three datasets; a protein–protein interaction (PPI) network was subsequently constructed and analyzed using Cytoscape. We then selected *NCAPG* for validation because of its poor prognosis and because it has not been sufficiently studied in the context of LUAD. Immunohistochemical analysis was used to detect the expression of NCAPG in LUAD tissues, and the relationships between NCAPG and clinical parameters were analyzed. In vitro and in vivo experiments were conducted to verify the role of NCAPG in LUAD. Finally, we studied the specific mechanism of action of NCAPG in LUAD.

**Results:**

Through comprehensive analysis of the GSE43458, GSE75037, and The Cancer Genome Atlas databases, we identified 517 overlapping DEGs. Among them, *NCAPG* was identified as a hub gene. Immunohistochemical analysis revealed that NCAPG was strongly associated with the clinical stage, M-classification, and N-classification. Univariate and multivariate Cox regression analyses indicated that NCAPG was a prognostic risk factor for LUAD, while the in vitro experiments showed that NCAPG overexpression promoted proliferation, migration, invasion, and epithelial-mesenchymal transition. Furthermore, knockdown of NCAPG inhibited LUAD progression, both in vitro and in vivo. Mechanistically, NCAPG overexpression increased p-Smad2 and p-Smad3 expressions in the transforming growth factor β (TGF-β) signaling pathway. Additionally, rescue experiments indicated that TGF-β signaling pathway inhibitors could restore the effect of NCAPG overexpression in LUAD cells.

**Conclusions:**

NCAPG may promote proliferation and migration via the TGF-β signaling pathway in LUAD.

**Supplementary Information:**

The online version contains supplementary material available at 10.1186/s12935-021-02138-w.

## Background

Lung cancer has the highest case fatality rate among all cancers because of uncontrolled proliferation and early metastasis of cancer cells in the lung tissue [1, 2]. Lung cancer includes small and non-small cell lung carcinoma; non-small cell lung cancer includes adenocarcinoma, squamous cell carcinoma, and large cell carcinoma, accounting for approximately 85% of all lung cancer cases [3]. The survival of patients with lung adenocarcinoma (LUAD) has increased with the advent of combined surgery, radiotherapy, and chemotherapy; however, the survival rate remains poor, as many patients are diagnosed at an advanced stage [4, 5]. Hence, it is essential to identify the underlying molecular mechanisms and significant prognostic biomarkers of LUAD.

Through the integrated analysis of the GSE43458, GSE75037, and The Cancer Genome Atlas (TCGA) databases for LUAD, we identified 517 overlapping differentially expressed genes (DEGs). Hub genes were selected by constructing a protein–protein interaction (PPI) network; we then analyzed the gene ontology (GO) and Kyoto Encyclopedia of Genes (KEGG) pathways of these genes. Among the hub genes, non-SMC condensin I complex subunit G (NCAPG)—a chromosome-condensed protein related to mitosis, with a relative molecular weight of approximately 114 kDa—was selected to be associated with a poor prognosis.

Previous researchers have found that NCAPG plays a vital role in condensin activation and the stabilization of chromosomes during mitosis [6], and increasing evidence shows that NCAPG overexpression is positively correlated with liver [7–10], gastric [11, 12], breast [13, 14], and renal cancer [15]; however, the functional role of NCAPG in LUAD remains unclear. To verify this, we conducted in vitro and in vivo experiments to determine whether NCAPG affects the malignant behavior of LUAD.

## Methods

### Biological information analysis

GSE43458 (including 80 LUAD and 30 adjacent normal lung tissues) and GSE75037 (including 83 LUAD and 83 adjacent normal lung tissues) data were obtained from the GEO database; the GEO2R method of analysis was performed on the two GEO datasets. In addition, RNA sequencing data from the TCGA LUAD cohort were downloaded, containing 478 LUAD and 57 adjacent normal lung tissues. The “limma” package in R (version 3.6.2; R Foundation for Statistical Computing, Vienna, Austria) was utilized to identify the DEGs between LUAD and normal lung tissue samples; genes—with “adjusted *P*-value < 0.05” and “|logFC| ≥ 1” as basic parameters—were selected as DEGs from each dataset.

The R package, clusterProfiler [16]—able to analyze and visualize the functional profiles of the genome coordinates—was used to perform GO functional enrichment and KEGG pathway enrichment analysis. PPI analysis was performed using STRING (https://string-db.org/) to identify DEGs; the results were visualized using Cytoscape [17], while hub genes were identified using CytoHubba [18] (degree ≥ 40).

To explore the possible molecular mechanisms of NCAPG in lung cancer cell lines, we downloaded and compiled RNA-seq data from 80 LUAD cell lines from the Cancer Cell Line Encyclopedia website [19]. We performed gene set enrichment analysis (GSEA) [20] on the data of the LUAD cell lines to further explore the signaling pathways of NCAPG.

The Comparative Toxicogenomics Database (CTD, http://ctdbase.org/) [21] is a website that helps improving understanding of the chemical-gene-disease relationship. In CTD, the links between chemical-gene-disease are extracted from the literature. Furthermore, a network between chemotherapeutic drugs and NCAPG was constructed based on CTD and visualized using Cytoscape.

### Patients and tissue samples

We collected 292 paraffin-embedded primary LUAD cancer tissues from patients at the Fujian Provincial Hospital, Fujian Medical University. Samples were collected between January 2010 and January 2021. The Ethics Committee of Fujian Provincial Hospital approved the use of all samples (K2021-04-092). Patients with a precise diagnosis and complete clinical information, who had not previously received radiation or chemotherapy, were included.

### Immunohistochemical analysis

The samples were prepared with a thickness of 0.5 μm, dewaxed, rehydrated, and incubated with NCAPG antibodies (1:200; Abcam, Cambridge, UK) and Ki67 antibodies (1:200; Abcam) in a wet box at 4 °C. After removing the antibodies, the samples were incubated with HRP-conjugated secondary antibodies; diaminobenzidine (1:50) was then added, and the cells were washed with phosphate-buffered saline (PBS) after 1 min.

Five fields of view were randomly selected for each slice and scored using a double-blind method by more than two experienced pathologists; the scores were based on the percentage of stained cells and degree of staining. Regarding the number of pigmented cells, 0 indicated that 0–10% of cells were stained, and 1, 2, 3, and 4 respectively indicated that 11–25%, 26–50%, 51–75%, and > 75% of cells were stained. Regarding the degree of staining, 0 indicated no staining, and 1, 2, and 3 respectively indicated light yellow, yellow, and brownish yellow staining. The two scores were multiplied to obtain a final quantification for each sample. Staining intensity was graded as negative for scores 0–3, weakly positive for scores 4–6, moderately positive for scores 7–9, and strongly positive for scores 10–12. Negative and weakly positive scores were considered to indicate low expression; conversely, moderately and strongly positive scores were considered to indicate high expression.

### Cell culture and infection

The human bronchial epithelial and LUAD cell lines—BEAS-2B, and PC9, A549, H827, H1299, and H1975—were purchased from the cell bank of the Chinese Academy of Sciences in Shanghai, China; they were then cultured in RMPI-1640 medium with 10% fetal bovine serum and incubated at 37 °C with 5% CO_2_. Cell lines were authenticated to be free of mycoplasma contamination. PC9 and A549 cells were infected with lentiviruses harboring NCAPG overexpression. NCAPG-targeting short hairpin RNA (shRNA) was synthesized by Hanheng Biology Co., Ltd. (Shanghai, China). The target sequences of shRNA were as follows: sh-NCAPG 1#: 5′-GCTGTCAGAAAGCTGGCTTAT-3′ and sh-NCAPG 2#: 5′-GCTGAAACATTGCAGAAATGT-3′. RepSox was purchased from Selleck (Shanghai, China).

### Western blot assay

The tissues and cells were lysed with a protease inhibitor cocktail for 20 min and then centrifuged to remove the supernatant. Protein concentration was quantified using a BCA kit; 30 μg of lysate from each sample was then separated by SDS–polyacrylamide gel electrophoresis, then blotted onto polyvinylidene fluoride membranes. Next, the primary antibody was added after sealing with 3% bovine serum albumin. Detailed information regarding the antibodies used in this study is listed in Additional file 1: Table S1. After washing the primary antibody with PBS, the corresponding HRP-linked secondary antibody was added; enhanced chemiluminescence was added for detection.

### Cell Counting Kit 8 (CCK8) assay

CCK8 (Beyotime Biotechnology, Shanghai, China) was used to evaluate cell proliferation. After lentivirus infection, CCK8 reagent was added to the culture medium; the absorbance at 450 nm was measured after 24, 48, 72, and 96 h.

### Clone formation experiment

Cells were trypsinized and inoculated in a six-well plate at approximately 1000 cells/well. The plate was frequently observed, and the culture was stopped when clones were visible; the plate was then fixed with methanol, stained with crystal violet, and quantified.

### Wound healing assay

Cells were cultured in a six-well plate at a cell density of 5 × 10^5^ cells/well. When the cells reached 90% confluence, a straight line was vertically drawn at the bottom of the well using the tip of a 10 μL pipette. Debris was removed and cultured in serum-free RPMI-1640 medium, and photographs were acquired at 0 and 48 h.

### Transwell migration assay

In the cell migration experiment, 200 μL of cell suspension (1 × 10^5^/mL) with serum-free RPMI-1640 medium was added to the upper well, while 600 μL medium containing 20% fetal bovine serum without cells was added to the lower chamber. After 24 h, the upper chamber was carefully removed, washed, fixed with paraformaldehyde, and stained with crystal violet. The non-migrating cells in the upper chamber were then gently wiped off with a cotton swab; photographs were subsequently obtained. Five fields of view were randomly selected for counting.

In the invasion experiment, the upper chamber was precoated with Matrigel (BD Biosciences, San Jose, CA) before the cells were added; subsequent steps were consistent with those of the migration experiments.

### Xenograft tumor model

Four- to five-week-old male BABL/c nude mice (14–18 g) were purchased from Slack Laboratory Animal Co., Ltd. (Shanghai, China). After lentivirus infection, PC9 cells (5 × 10^6^)—including sh-Control, sh-NCAPG 1#, and sh-NCAPG 2#—were injected into the right flanks of mice. Tumors were measured every 7 days, and their volumes were calculated as follows: (length × width^2^)/2. On day 28, the tumors were harvested for immunohistochemical analyses.

### Statistical analysis

Statistical analyses were performed using SPSS 22.0 (IBM Corp., Armonk, NY). The association between gene expression and clinicopathological characteristics was analyzed using the chi-squared test, while the Kaplan–Meier method was used for the survival analysis. In addition, differences between two groups were analyzed using Student's t-test, while differences among three or more groups were analyzed using one-way analysis of variance; statistical significance was set at p < 0.05. The assays were performed independently at least three times.

## Results

### Identification of DEGs in LUAD

**Fig. 1:**
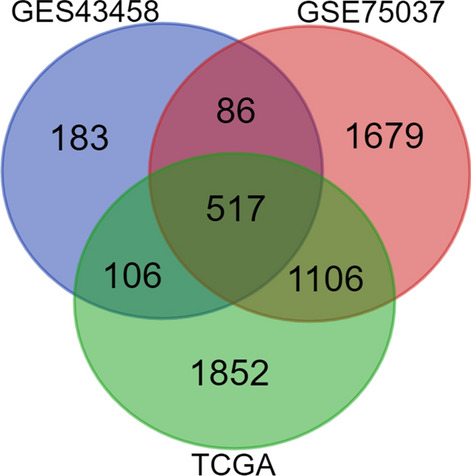
Overlapping differentially-expressed genes in the three datasets

**Fig. 2:**
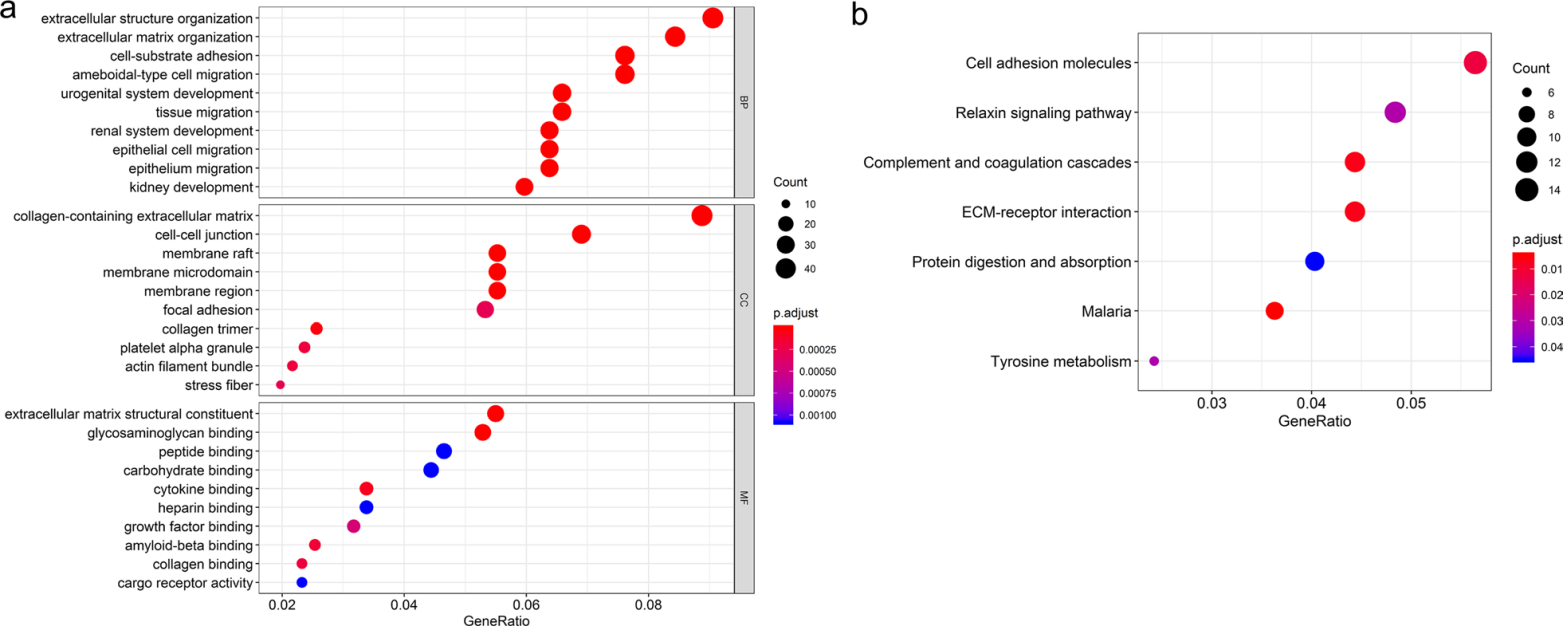
GO and KEGG enrichment of 517 overlapping differentially-expressed genes. **a** The enriched GO terms consisted of biological-process, cell-component, and molecular-function ontologies. **b** The KEGG pathway was mainly enriched with seven pathways. *ECM* extracellular matrix, *GO* gene ontology, *KEGG* Kyoto Encyclopedia of Genes

DEGs were determined by comparing LUAD and adjacent lung tissue samples. We identified 517 DEGs (Fig. 1, Additional file 2: Table S2) with an adjusted p-value < 0.05, and |log FC| ≥ 1. GO functional, and KEGG pathway enrichment analyses were performed to analyze the functions and mechanisms of common DEGs. The enriched GO terms included biological-process (BP), cell-component (CC), and molecular-function (MF) ontologies (Fig. 2a). BP analysis was enriched in the extracellular matrix organization, extracellular structure organization, cell-substrate adhesion, and others. CC analysis was mainly enriched in collagen-containing extracellular matrix, cell–cell junctions, membrane raft, and others. MF analysis was mainly enriched in extracellular matrix structural constituents, glycosaminoglycan binding, cytokine binding, among others. Additionally, the KEGG pathway (Fig. 2b) was mainly enriched in several pathways, including the relaxin signaling pathway, cell adhesion molecules, complement, and coagulation cascades.

### NCAPG identified as a targeted gene associated with poor prognosis

**Fig. 3:**
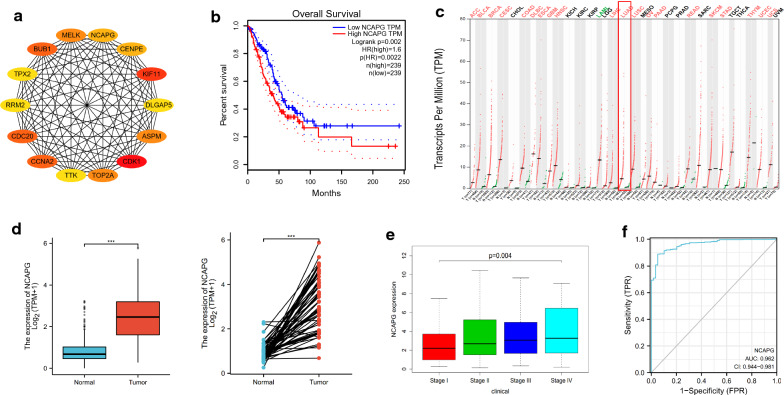
*NCAPG* was identified as a targeted gene associated with poor prognosis. **a** The top 14 genes were screened using Cytoscape according to the CytoHubba ranking methods. **b** High expression of NCAPG was associated with poor overall survival. **c** NCAPG was upregulated in most cancers. **d** NCAPG was upregulated in tissue with lung adenocarcinoma in the TCGA cohort. **e** Increased NCAPG expression was associated with an advanced clinical TNM stage in the TCGA database. **f** The receiver operating characteristic area under the curve was 0.962 (95% CI 0.944–0.981; p < 0.001). *FPR* false positive rate, *TPM* transcripts per million, *TPR* true positive rate

STRING was used to construct a PPI network from overlapping genes to identify the most important genes (Additional file 3: Fig. S1). The top 14 genes were screened using Cytoscape according to the CytoHubba ranking method (Fig. 3a, Table 1). GEPIA (http://gepia.cancer-pku.cn/) [22] was employed to study the prognosis of these hub genes, and it was found that high expression was associated with poorer overall survival (Additional file 4: Fig. S2). NCAPG represents one of these genes (Fig. 3b), which has not been well studied in LUAD; therefore, this gene was selected for further analysis. Furthermore, NCAPG expression was upregulated in most cancers (Fig. 3c).

**Table 1:** Top 14 hub genes with higher degree

Rank	Gene symbol	Gene description	Degree
1	CDK1	Cyclin Dependent Kinase 1	45
2	KIF11	Kinesin Family Member 11	44
3	CDC20	Cell Division Cycle 20	43
3	CCNA2	Cyclin A2	43
3	BUB1	BUB1 Mitotic Checkpoint Serine/Threonine Kinase	43
6	MELK	Maternal Embryonic Leucine Zipper Kinase	42
6	TOP2A	DNA Topoisomerase II Alpha	42
6	ASPM	Assembly Factor For Spindle Microtubules	42
9	NCAPG	Non-SMC Condensin I Complex Subunit G	41
9	CENPE	Centromere Protein E	41
11	RRM2	Ribonucleotide Reductase Regulatory Subunit M2	40
11	DLGAP5	DLG Associated Protein 5	40
11	TTK	TTK Protein Kinase	40
11	TPX2	TPX2 Microtubule Nucleation Factor	40

By analyzing the expression of NCAPG in patients with LUAD through the TCGA database, we found that NCAPG expression was upregulated (p < 0.001, Fig. 3d) in LUAD tissue samples compared with adjacent normal tissue samples; additionally, increased NCAPG expression was associated with advanced clinical TNM stages (Fig. 3e). The receiver operating characteristic area under the curve was 0.962 (95% CI 0.944–0.981; p < 0.001; Fig. 3f), indicating that NCAPG has high sensitivity and specificity for the diagnosis of LUAD.

### High NCAPG expression was associated with poor prognosis

**Fig. 4:**
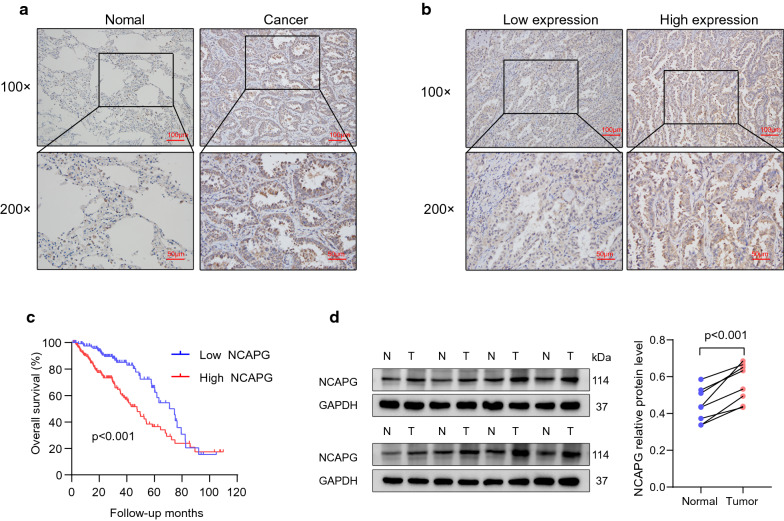
High NCAPG expression was associated with poor prognosis in our cohort. **a** Immunohistochemical staining revealed that NCAPG appears brown in lung adenocarcinoma (LUAD) tissue. **b** Representative photographs of low and high expression of NCAPG in LUAD tissue. **c** Kaplan–Meier analysis indicated that patients with LUAD and higher NCAPG expression had shorter survival. **d** Western blot analysis showed that NCAPG was overexpressed in human primary LUAD tissues compared with adjacent lung tissues

To verify the expression of NCAPG in LUAD, immunohistochemical analyses were used to detect the expression of NCAPG in 292 cases of paraffin-embedded LUAD tissues. Immunohistochemical staining showed that NCAPG appeared brown in the LUAD tissues (Fig. 4a).

Table 2 lists the demographics, clinicopathological characteristics, and tumor characteristics of the 292 patients with LUAD, including 142 female (48.6%) and 150 male (51.4%) patients aged 33–86 years. Postoperative pathological staging identified 150 cases (51.4%) at stage I, 72 cases (24.7%) at stage II, 51 cases (17.5%) at stage III, and 19 cases (6.5%) at clinical stage IV of the disease. The NCAPG protein level was overexpressed in 164 (56.2%) samples, and low in 128 (43.8%) samples (Fig. 4b).

**Table 2:** Clinicopathological characteristics of patient samples and expression of NCAPG in LUAD

Clinical characteristics	Number of cases (%)
Age (y)	
≥ 65	160 (54.8)
< 65	132 (45.2)
Sex	
Male	150 (51.4)
Female	142 (48.6)
Clinical stage	
I	150 (51.4)
II	72 (24.7)
III	51 (17.5)
IV	19 (6.5)
T classification	
T1	88 (30.1)
T2	164 (56.2)
T3	24 (8.2)
T4	16 (5.5)
N classification	
N0	184 (63.0)
N1	63 (21.6)
N2	44 (15.1)
N3	1 (0.3)
Metastasis	
No	273 (93.5)
Yes	19 (6.5)
Expression of NCAPG	
Low expression	128 (43.8)
High expression	164 (56.2)

T for extent of the primary tumor; N for involvement of lymph nodes

T1: Tumor ≤ 3 cm; T2: Tumor > 3 but ≤ 5 cm or tumor involving visceral pleura, main bronchus (not carina), atelectasis to hilum; T3: Tumor > 5 but ≤ 7 cm or invading chest wall, pericardium, phrenic nerve or or separate tumor nodule(s) in the same lobe; T4: Tumor > 7 cm or tumor invading: mediastinum, diaphragm, heart, great vessels, recurrent laryngeal nerve, carina, trachea, esophagus, spine or tumor nodule(s) in a different ipsilateral lobe

N0: No regional node metastasis; N1: Metastasis in ipsilateral pulmonary or hilar nodes; N2: Metastasis in ipsilateral mediastinal/subcarinal nodes; N3: Metastasis in contralateral mediastinal/hilar, or suprclavicular nodes

The relationship between NCAPG and the clinicopathological parameters of LUAD was also analyzed. NCAPG expression was strongly correlated with N-classification (p = 0.006), M-classification (p = 0.011), and clinical stage (p = 0.004), as shown in Table 3; no correlations were observed regarding age (p = 0.407), sex (p = 0.556), or T-classification (p = 0.091).

**Table 3:** Association between NCAPG expression and clinicopathologic characteristics of LUAD patients

Clinical characteristics	NCAPG		p-value
	Low expression	High expression	
Age (y)			
≥ 65	74	86	0.407
< 65	54	78	
Sex			
Male	63	87	0.556
Female	65	77	
Clinical stage			
I	79	71	0.004
II	28	44	
III & IV	21	49	
T classification			
T1	47	41	0.091
T2	66	98	
T3 & T4	15	25	
N classification			
N0	92	92	0.006
N1 & N2 & N3	36	72	
Metastasis			
No	125	148	0.011
Yes	3	16	

T for extent of the primary tumor; N for involvement of lymph nodes

T1: Tumor ≤ 3 cm; T2: Tumor > 3 but ≤ 5 cm or tumor involving visceral pleura, main bronchus (not carina), atelectasis to hilum; T3: Tumor > 5 but ≤ 7 cm or invading chest wall, pericardium, phrenic nerve or or separate tumor nodule(s) in the same lobe; T4: Tumor > 7 cm or tumor invading: mediastinum, diaphragm, heart, great vessels, recurrent laryngeal nerve, carina, trachea, esophagus, spine or tumor nodule(s) in a different ipsilateral lobe

N0: No regional node metastasis; N1: Metastasis in ipsilateral pulmonary or hilar nodes; N2: Metastasis in ipsilateral mediastinal/subcarinal nodes; N3: Metastasis in contralateral mediastinal/hilar, or suprclavicular nodes

Kaplan–Meier analysis indicated that among patients with LUAD, those with higher NCAPG expression had shorter survival than those with lower NCAPG expression (p < 0.001; Fig. 4c), while univariate and multivariate Cox regression analyses showed that NCAPG was an independent prognostic factor in patients with LUAD (Table 4). Furthermore, we analyzed eight paired tumor and non-cancerous lung tissues to detect the expression of NCAPG. Western blot analysis showed that NCAPG was over expressed in human primary LUAD tissues compared with adjacent lung tissues (Fig. 4d).

**Table 4:** Univariate and multivariate Cox regression analyses of various prognostic parameters in patients with LUAD

Parameter	Univariate analysis			Multivariate analysis		
	HR	95% CI	P	HR	95% CI	P
Age (y)	1.030	0.697–1.523	0.882			
Sex	0.963	0.654–1.418	0.848			
Clinical stage	1.834	1.528–2.201	< 0.001	2.200	1.092–2.591	0.002
T classification	1.645	1.304–2.076	< 0.001	1.121	0.861–1.459	0.396
N classification	1.931	1.559–2.393	< 0.001	0.977	0.656–1.454	0.908
Metastasis	2.136	1.135–4.020	0.019	0.358	0.114–1.130	0.080
NCAPG	2.048	1.348–3.111	< 0.001	1.682	1.092–2.591	0.018

T for extent of the primary tumor; N for involvement of lymph nodes

### NCAPG promoted proliferation and metastasis in LUAD cells

**Fig. 5:**
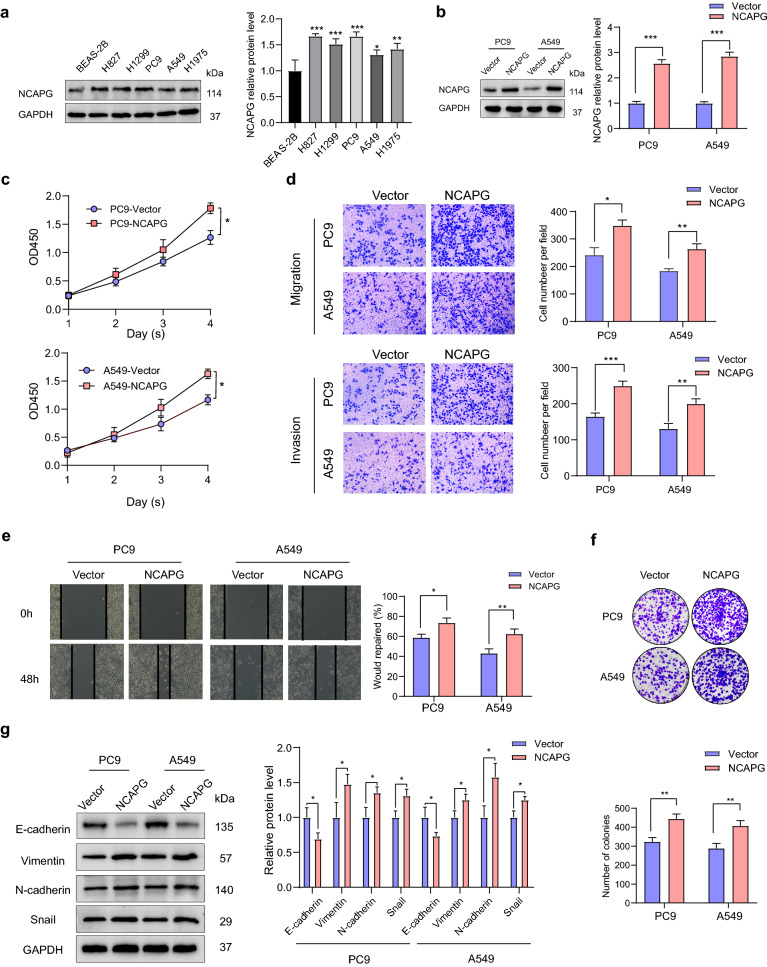
Effect of NCAPG overexpression on PC9 and A549 cell lines in vitro. **a** Western blot analysis of NCAPG expression in BEAS-2B and lung adenocarcinoma cell lines. **b** Western blot analysis of PC9 and A549 cells infected with vectors and NCAPG overexpression lentiviruses. **c**–**f** PC9 and A549 cells infected with vectors and NCAPG overexpression lentiviruses were subjected to CCK8, transwell, wound healing, and clone formation assays. **g** Expression of epithelial-mesenchymal transition-related markers (E-cadherin, N-cadherin, vimentin, and Snail) in PC9 and A549 cells. *p < 0.05, **p < 0.01, ***p < 0.001

**Fig. 6:**
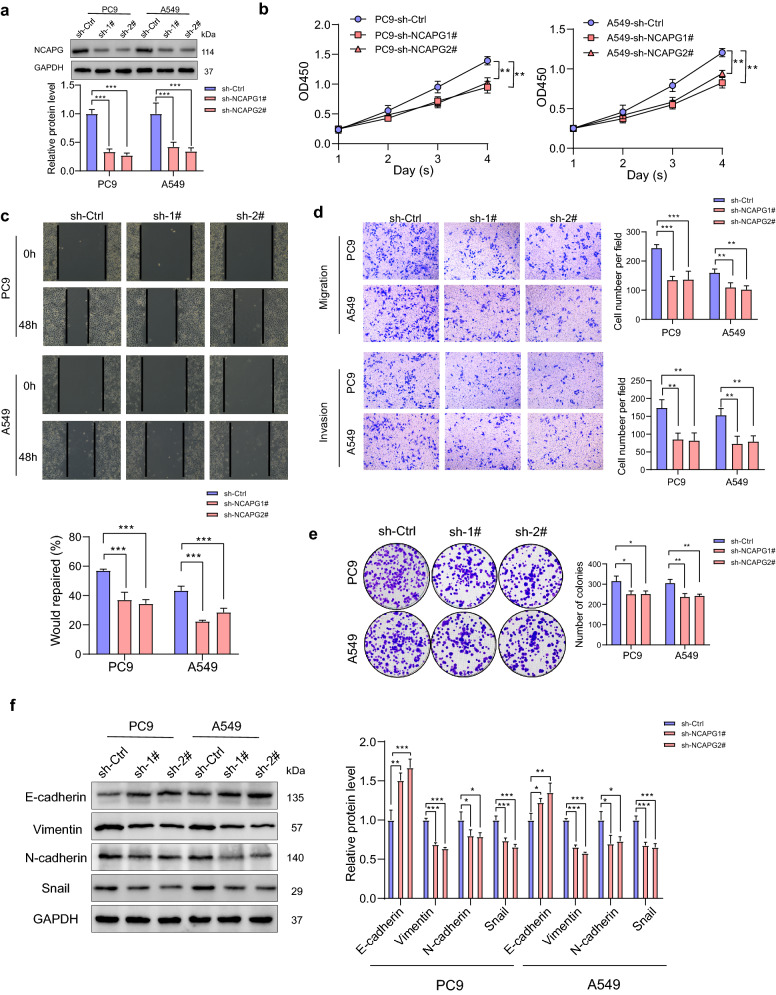
Effect of NCAPG knocking down on PC9 and A549 cell lines in vitro. **a** Western blot analysis of PC9 and A549 cells infected with sh-Ctrl, sh-NCAPG1#, and sh-NCAPG2# lentiviruses. **b**–**e** PC9 and A549 cells infected with sh-Ctrl, sh-NCAPG1#, and sh-NCAPG2# lentiviruses were subjected to CCK8, transwell, wound healing, and clone formation assays. **f** Expression of epithelial–mesenchymal transition-related markers (E-cadherin, N-cadherin, vimentin, and Snail) in PC9 and A549 cells. *p < 0.05, **p < 0.01, ***p < 0.001. *Ctrl* control

Through western blot analyses, we verified that NCAPG expression in LUAD cell lines was upregulated compared with that in BEAS-2B cell lines (Fig. 5a). To further validate the role of NCAPG in LUAD cells, we ectopically overexpressed NCAPG in PC9 and A549 LUAD cells (Fig. 5b). CCK8 assays, colony formation, wound healing, and transwell assays were performed to identify the role of NCAPG in the progression, invasion, and migration of LUAD (Fig. 5c–f); interestingly, it was found to induce cell progression, migration, and invasion in PC9 and A549 cell lines. Additionally, we constructed cells with stable *NCAPC* knockdown using shRNAs (Fig. 6a); silencing of *NCAPG* led to the inhibition of cell proliferation, invasion, and migration (Fig. 6b–e). Epithelial–mesenchymal transition (EMT) marker genes were further detected with western blot analysis. We found that when NCAPG was overexpressed, the expression of N-cadherin, vimentin, and Snail increased, while the expression of E-cadherin decreased (Fig. 5g); conversely, when *NCAPG* was knocked down, the expression of N-cadherin, vimentin, and Snail decreased, while the expression of E-cadherin increased (Fig. 6f). These results indicate that NCAPG plays a key role in the progression of LUAD.

### NCAPG modulated the tumorigenesis of LUAD

**Fig. 7:**
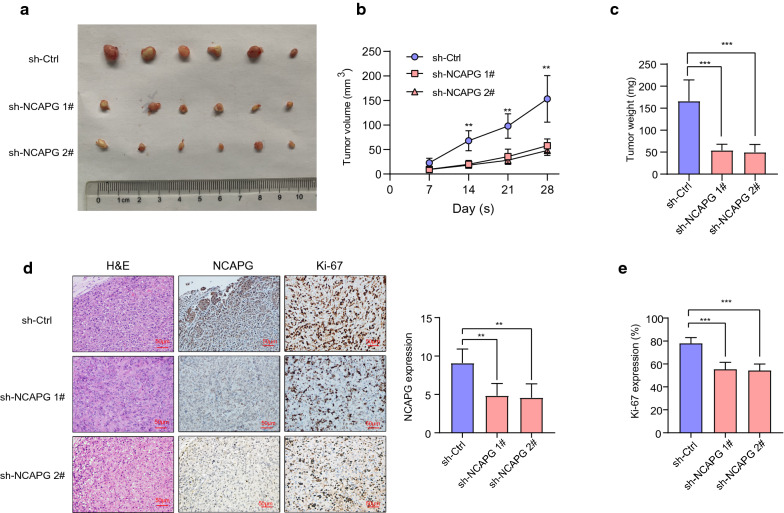
Effect of NCAPC knockdown on the PC9 cell line in vivo. **a** Photographs of xenograft tumors. **b** Average weight of xenograft tumors. **c** Growth curve of xenograft tumors. **d** Hematoxylin and eosin staining and immunohistochemical staining of NCAPG and Ki-67 in xenograft tumors. **e** Quantitation of Ki-67 staining. *p < 0.05, **p < 0.01, ***p < 0.001. *Ctrl* control

To verify the results of the in vivo experiments, we used PC9 cells in male BALB/C nude mice to establish a xenograft model. As shown in Fig. 7a, compared with the cells infected with the vector, the ability of *NCAPG*-silenced cells to form tumors in nude mice was significantly reduced, indicated by the weight and growth curve of the tumor (Fig. 7b, c). NCAPG was detected in tumors using immunohistochemical staining, revealing that tumors formed by sh-NCAPG cells were significantly lower than those of sh-Ctrl cells (Fig. 7d). In tumors formed by sh-NCAPG cells, the expression of Ki-67 was decreased compared with the sh-Ctrl group (Fig. 7e). Ultimately, NCAPG was found to play an oncogenic role in LUAD.

### NCAPG promoted LUAD progression and proliferation through the transforming growth factor β (TGF-β) signaling pathway

**Fig. 8:**
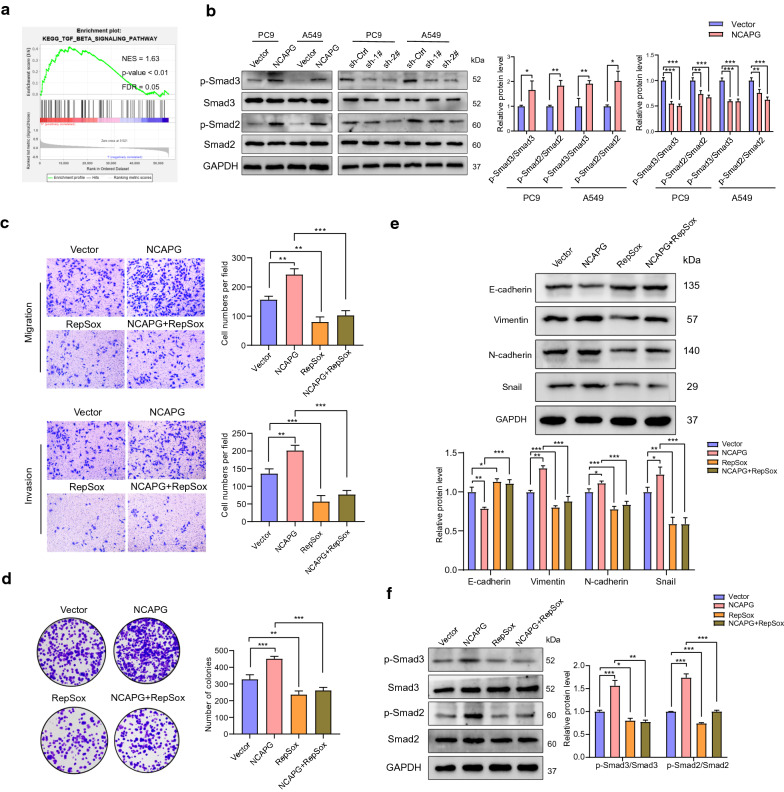
NCAPG promotes lung adenocarcinoma progression and proliferation through the TGF-β signaling pathway. **a** GSEA analysis revealed that the abnormal expression of NCAPC is significantly related to the TGF-β signaling pathway. **b** Western blot analysis of p-Smad3, Smad3, p-Smad2, and Smad2. **c** Transwell assays were performed to investigate the role of the TGF-β signaling pathway in the migration and invasion ability of NCAPC-overexpressed A549 cells. **d** Clone formation assays were performed to investigate the role of the TGF-β signaling pathway in the proliferation ability of NCAPC-overexpressed A549 cells. **e** Western blot analysis of E-cadherin, N-cadherin, vimentin, and Snail was performed to determine the role of the TGF-β signaling pathway in the epithelial–mesenchymal transition of NCAPG-overexpressed A549 cells. **f** Western blot analysis of p-Smad3, Smad3, p-Smad2, and Smad2 was performed to determine the effect of RepSox after NCAPG overexpression. *p < 0.05, **p < 0.01, ***p < 0.001

GSEA enrichment analysis was performed to explore the pathogenic mechanism of NCAPG in LUAD, revealing that the abnormal expression of NCAPG was related to the TGF-β signaling pathway (Fig. 8a). We detected the main proteins involved in the TGF-β signaling pathway, and the results are shown in Fig. 8b. After overexpressing NCAPG, the phosphorylation levels of Smad2 and Smad3 increased, whereas the total Smad2 and Smad3 levels remained constant. After *NCAPG* knockdown, the phosphorylation levels of Smad2 and Smad3 significantly decreased; however, no change in the total Smad2 and Smad3 levels was observed.

### Inhibiting TGF-β reduced progression of LUAD induced by overexpressing NCAPG

To confirm that NCAPG functions via the TGF-β signaling pathway, the TGF-β inhibitor RepSox was added to the culture medium after NCAPG overexpression. Colony formation and transwell assays were used to determine proliferation, migration, and invasion ability. Interestingly, cell proliferation, migration, and invasion ability were eliminated in the NCAPG + RepSox group (Fig. 8c, d); additionally, western blot analysis showed that NCAPG + RepSox inhibited EMT induced via NCAPG overexpression, characterized by upregulation of E-cadherin, and downregulation of N-cadherin, vimentin, and Snail (Fig. 8e). Simultaneously, NCAPG + RepSox weakened the activation of the TGF-β/Smad signaling pathway through overexpression of NCAPG (Fig. 8f). These results suggest that inhibition of TGF-β reduces the tumorigenic effect of NCAPG in LUAD.

### Identify chemotherapeutic drugs targeting NCAPG

**Fig. 9:**
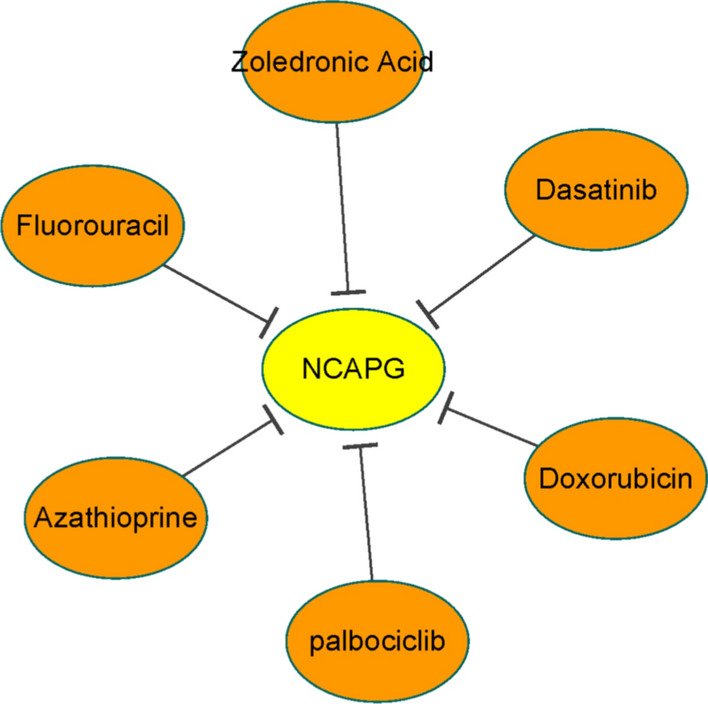
The network of NCAPG and chemotherapeutic drugs constructed using CTD. *CTD* comparative toxicogenomics database; vertical arrow: chemotherapeutic drugs decreased the expression of NCAPG

To gain further insight into the gene-targeted therapy, a network between chemotherapeutic drugs and NCAPG was constructed based on CTD. As shown in Fig. 9, the network showed that fluorouracil, azathioprine, palbociclib, doxorubicin, dasatinib, and zoledronic acid could reduce the expression of NCAPG.

## Discussion

Data mining can help us obtain more information regarding the mechanisms of tumor occurrence and development. Many oncogenes and tumor suppressor genes can be evidenced by multi-omics analyses from tumor tissues, and these genes are closely related to the prognosis of patients. By examining the copy number changes and mutation of protein-coding genes in 100 breast cancer tissues, Stephens et al. [23] found at least 40 oncogenes related to poor prognosis. Liu et al. [24] synthesized data from 2778 patients from 15 public data sets to identify reliable biomarkers for the prognosis and distant metastasis risk of colorectal cancer. Bioinformatics analyses were applied in our study by integrating three databases, i.e., GSE43458, GSE75037, and TCGA, and 517 DEGs were identified. Among the common DEGs, 14 hub genes were screened using CytoHubba. NCAPG was found upregulated and associated with poor survival in patients with LUAD.

The expression of NCAPG has been reported to be associated with tumor progression. Gong et al. [7] performed immunohistochemical analyses in 90 hepatocellular carcinoma (HCC) and adjacent tissues; the results indicated that the expression of NCAPG was higher in 85 HCC tissues than in non-HCC tissues. The group’s in vitro experiments demonstrated that NCAPG promoted the proliferation and metastasis of HCC cells by activating the PI3K/AKT/FOXO4 pathway. Another study reported that NCAPG was significantly overexpressed in trastuzumab-resistant HER2+ breast cancer samples and that the upregulation of NCAPG was associated with poor survival and recurrence in patients with HER2+ breast cancer [13]; thus, to further determine whether NCAPG promotes LUAD progression, we designed a series of studies. Our findings provide evidence that NCAPG is both significantly upregulated in LUAD and associated with poor survival. Additionally, NCAPG overexpression promoted LUAD cell proliferation, invasion, and migration; therefore, we speculate that *NCAPG* may be an oncogene in LUAD and could serve as a potential biological target for the diagnosis of LUAD.

EMT can induce the escape and apoptosis of tumor cells, as well as promote migration and infiltration; once tumor cells undergo EMT, the possibility of metastasis increases [25]. The most significant changes in EMT are the decrease or absence of E-cadherin and increase of N-cadherin, vimentin, and Snail. After EMT is activated, the tumor epithelial cells lose cell polarity and intercellular adhesion and gain migratory and invasive characteristics; they then change to mesenchymal cells, thereby promoting tumor cell metastasis. Currently, there is a general consensus that EMT is critical for modulating drug resistance, cancer invasion, and metastasis [26, 27]. Our study found that the overexpression of NCAPG led to upregulation of N-cadherin, vimentin, and Snail, while E-cadherin was inhibited, proving that NCAPG promoted EMT in LUAD cells. Conversely, the downregulation of NCAPG led to a decrease in N-cadherin, vimentin, and Snail, and an increase in E-cadherin. We believe that NCAPG aggravates migration and invasion by improving EMT in LUAD.

We found that NCAPG was positively correlated with the TGF-β signaling pathway—as shown by GSEA—and it is well accepted that EMT can be induced by the TGF-β signaling pathway [28, 29]. TGF-β is an inhibitory factor in precancerous cells, while in cancer cells, it plays a role in promoting metastasis. Tumor cells can secrete large amounts of TGF-β, promote tumor invasion and metastasis through the autocrine and paracrine pathways, and induce EMT, causing epithelial tumor cells to acquire an aggressive mesenchymal phenotype [30]. At the same time, secretion of intercellular adhesion molecules and metalloproteinases changes cause tumor metastasis [31].

**Fig. 10:**
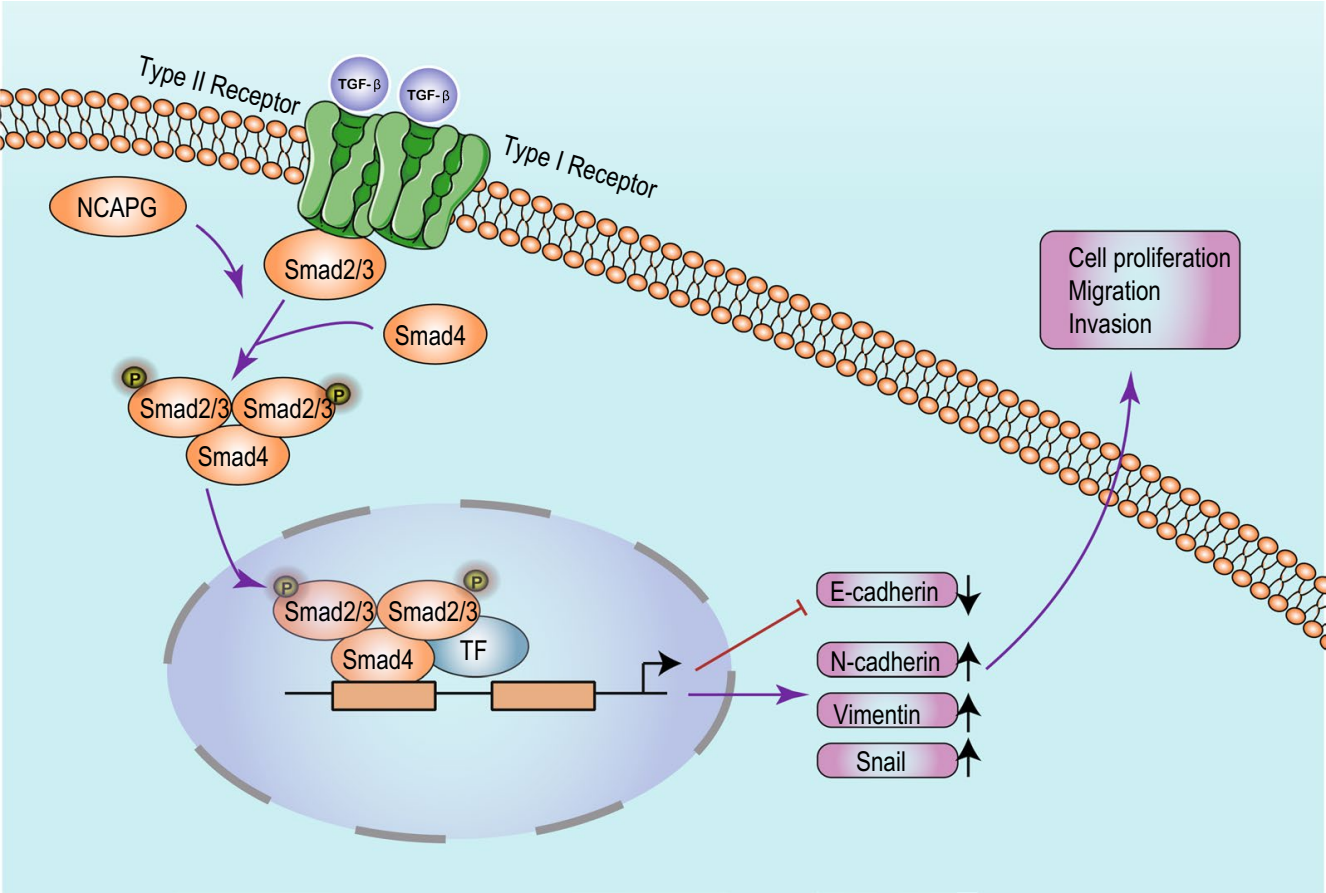
A proposed model illustrating the function and mechanism of NCAPG in LUAD progression. *TF* transcription factors

Smad2 and Smad3 were found to mediate the classical TGF-β/Smad signaling pathway. In the TGF-β signaling pathway, the TGF-β superfamily ligands bind to membrane TβRII, subsequently phosphorylating TβRI and forming complexes. The activated receptor complex further activates Smad2 and Smad3, and phosphorylated Smad2/Smad3 binds to Smad4; the Smad4 complex then enters the nucleus and regulates the expression of target genes with other transcription factors [32, 33]. In this study, we found that NCAPG promoted the progression of LUAD by positively regulating the TGF-β pathway; additionally, a TGF-β pathway inhibitor could reverse the effect of NCAPG overexpression on the progression of LUAD. It can therefore be concluded that NCAPG partially promotes LUAD progression through the TGF-β pathway (Fig. 10).

In order to explore potential gene-targeted treatments against LUAD with NCAPG overexpression, we used CTD to construct a gene-drug network. Based on this network, we found that fluorouracil, azathioprine, palbociclib, doxorubicin, dasatinib, and zoledronic acid can reduce the expression of NCAPG, indicating that these drugs might be a therapeutic alternative for these patients. For instance, several studies have reported dasatinib as a growth inhibitor of lung cancer in vitro and in vivo [34, 35]. Pabocinil, mainly used for breast cancer treatment, was reported to be a potential therapeutic agent in patients with non-small cell lung cancer [36]. However, whether LUAD patients with overexpressing NCAPG can benefit from the use of these drugs still needs further experimental evidence.

There are some limitations to our study; first, further experiments are needed to study the specific mechanism by which NCAPG induces the activation of the TGF-β signaling pathway and EMT. Second, previous research on the progress of NCAPG in LUAD—especially its correlation with the TGF-β signaling pathway—is limited. To a certain extent, this also limits our exploration of its molecular mechanism; therefore, our findings provide a foundation for further research on the downstream mechanisms of NCAPG-induced LUAD progression.

## Conclusion

In summary, our study was the first to prove that *NCAPG* is an oncogene in LUAD. Moreover, NCAPG-induced LUAD may be related to the TGF-β pathway. These findings provide new insights into the role of NCAPG regarding the occurrence and development of LUAD, which may further our understanding of the biological behavior of NCAPG, as well as help establish a potential treatment for LUAD.

## Supplementary Information

**Additional file 1: Table S1.** Antibodies used in this study.

**Additional file 2: Table S2.** Overlapping differentially expressed genes in the three databases.

**Additional file 3: Figure S1.** PPI network of overlapping genes.

**Additional file 4: Figure S2.** Kaplan–Meier overall survival analysis of the top 14 genes in patients with LUAD.

## Data Availability

The data that support the findings of this study are openly available in The Cancer Genome Atlas (TCGA) data portal (https://tcga-data.nci.nih.gov/tcga/) and Gene Expression Omnibus (GEO) database (https://www.ncbi.nlm.nih.gov/geo/). The rest of the data are available from the corresponding author on reasonable request.

## Abbreviations

LUAD: Lung adenocarcinoma
NCAPG: Non-SMC condensin I complex, subunit G
TCGA: The Cancer Genome Atlas
DEG: Differentially expressed gene
PPI: Protein–protein interaction
GO: Gene ontology
BP: Biological process
CC: Cell component
MF: Molecular function
KEGG: Kyoto Encyclopedia of Genes
GSEA: Gene set enrichment analysis
CCK8: Cell Counting Kit 8
EMT: Epithelial-mesenchymal transition
HCC: Hepatocellular carcinoma
PBS: Phosphate buffered saline
shRNA: Short hairpin RNA
TGF-β: Transforming growth factor β
CTD: Comparative Toxicogenomics Database
