# Supplementary material for: NCAPG promotes the progression of lung adenocarcinoma via the TGF-β signaling pathway
Source: Cancer Cell Int. 2021 Aug 21;21:443. doi: 10.1186/s12935-021-02138-w (PMC8380402; doi:10.1186/s12935-021-02138-w)
Supplement: Supplementary file 1 — Additional file 1: Table S1. Antibodies used in this study. [file 12935_2021_2138_MOESM1_ESM.docx]

Table S1 Antibodies used in this study

| Protein | Cat no. | Company | Source | KD |
| --- | --- | --- | --- | --- |
| NCAPG | ab226805 | Abcam | Rabbit | 114 |
| E-cadherin | 3195 | CST | Rabbit | 135 |
| N-cadherin | 13116 | CST | Rabbit | 140 |
| Vimentin | 5741 | CST | Rabbit | 57 |
| Snail | 3879 | CST | Rabbit | 29 |
| Smad3 | 9523 | CST | Rabbit | 52 |
| p-Smad3 | 9520 | CST | Rabbit | 52 |
| Smad2 | 5339 | CST | Rabbit | 60 |
| p-Smad2 | 18338 | CST | Rabbit | 60 |
| GAPDH | 51332 | CST | Mouse | 37 |
