# Supplementary material for: NCAPG promotes the progression of lung adenocarcinoma via the TGF-β signaling pathway
Source: Cancer Cell Int. 2021 Aug 21;21:443. doi: 10.1186/s12935-021-02138-w (PMC8380402; doi:10.1186/s12935-021-02138-w)
Supplement: Supplementary file 2 — Additional file 2: Table S2. Overlapping differentially expressed genes in the three databases. [file 12935_2021_2138_MOESM2_ESM.docx]

Table S2 Overlapping differentially expressed genes in the three databases.

| DEGs |
| --- |
| ABCA3 XDH EDN1 CCNB1 MSRB3 AQP9 NOSTRIN CXCL13 A2M HIGD1B ANXA3 TEK COL1A1 GRK5 VGLL3 FOXM1 ZNF385B CDK1 BCHE TOX3 RSPH4A SPTBN1 TSPAN7 PPBP ACADL AHNAK KIF14 FHL1 KLF4 PLSCR4 NDRG2 TCF21 ARHGEF26 LDLR KIF4A METTL7B DNAJB4 NTN4 TMPRSS4 ENKUR SPAG6 OLR1 GLIPR2 AGER HOXA5 CD93 CLDN10 WIF1 TYMS FLI1 MELK GPX2 CLIC5 NPNT PROS1 CSRNP1 MME CCNA2 ICAM2 GBP4 GPM6A MMP1 WFS1 HSD17B13 DEPDC1 FAM83A LMO7 PPP1R15A STARD8 LRRC32 NRN1 SPAG5 OGN ESAM TACC1 C20orf85 KRT4 FMO3 CXCL2 SESN1 CCNB2 PRC1 CEACAM5 FUT3 GIMAP4 NECTIN4 PDE5A CPA3 ROBO4 COMP TNFRSF21 CDC45 TBX3 FAM184A SPTBN2 PDZD2 HBB GNG11 RHOJ FCRL5 SRPX SCN7A TSPAN18 CKAP2L CABYR SFTPC SULF1 MYL9 LPL EXO1 SOSTDC1 SBSPON GPC3 ADCY4 FPR2 CCBE1 PGM5 LMCD1 FOSB MARCO DCN COL11A1 S100A12 SLIT2 CX3CR1 FUT2 FZD4 PCOLCE2 UGT8 HORMAD1 AOX1 GKN2 CYP4B1 SMAD6 AGR2 RFX2 FAM216B XRCC2 KIF26B WWC2 CHRDL1 HSPB8 ITIH5 MMP13 NLRC4 MMRN2 ITLN1 DSP NDST1 ST6GALNAC5 JAM3 AKR1B10 ACVRL1 TIMP3 EML1 RERG EZH2 MUC16 HBEGF CRYAB CHEK1 KIF11 CD36 ETS2 ADGRL2 AQP4 CST1 ITGA8 IL1RL1 MCEMP1 SELE FAR2 KLF6 THBD ABCA8 TMEM47 GLB1L2 ZBTB16 EDNRB SLIT3 FAM167A MYH11 MATN3 DUSP1 GPRC5A GSDMC UACA ABI3BP PYCR1 TIE1 ARRB1 DAPK2 CDCA7 MS4A15 CDC20 GREM1 C7 WASF3 KDELR3 BUB1 F8 IL6 CPED1 PRICKLE2 TMEM100 KLRF1 COL3A1 KIAA1324L DPT ABCG2 PECAM1 TBX4 OCIAD2 MAOB HGD BMPR2 B3GALNT1 CDO1 ID4 CLEC14A ANOS1 C8B STIL NQO1 CD79A S1PR1 GIMAP7 SLC1A1 PSAT1 PTPN21 FOS SPOCK2 RNF144B RRM2 TOP2A LDB2 CAV1 HELLS CLEC1A JAM2 SPARCL1 ANKRD29 FGD5 ARHGAP44 IL7R TLR4 SOCS2 RBMS3 TMOD1 ST6GALNAC2 FGFBP2 ARHGEF6 TGFBR2 IL33 PGC CTNNAL1 FABP4 CRTAC1 FAM107A EGLN3 GPM6B AIM2 SGCE MMRN1 LCN2 CTHRC1 MAMDC2 FBXO32 CDKN3 SULT1C4 CDH5 SELENBP1 CENPF PTGIS FBLN5 PLCE1 FIBIN RTKN2 FRAS1 TPX2 PEBP4 C15orf48 IGF2BP3 ITLN2 SUSD2 DKK2 PAPSS2 KPNA2 ANLN CHRNA5 MS4A2 SLCO2A1 ADGRF1 LAMP3 STARD13 GPD1 LRRK2 AFAP1L1 SULT1C2 CLEC3B NEBL HLF GALNT18 NDNF PHACTR1 S100A3 ZFP36 C1orf198 MEIS1 GLDN VSIG1 ANKRD22 SELP CFAP43 SLC50A1 SPP1 SFTPD ADH1C HNF4G MSR1 RRAS TPPP3 MXRA5 ROPN1L RAMP3 PLPP2 SPRYD7 FHL5 TIMP1 ABCA12 PDK4 MMP12 LRRC36 HABP2 UBE2T SLC2A1 EPCAM ANGPTL1 EFEMP1 P2RY14 CACNA2D2 ECT2 MMP11 CDH3 EMCN ADARB1 CGNL1 CENPE CRABP2 TSPAN12 SASH1 SCGB1A1 CA4 PLA2G1B ADAM28 CHI3L2 LYVE1 P3H2 CEP55 AKAP12 CEMIP PHACTR2 FEZ1 LAD1 CBX7 PID1 RALGPS2 TFAP2A RAB11FIP1 DLGAP5 RAD51AP1 FLRT3 ZNF106 SLC39A8 MKI67 SHROOM4 TGFBR3 CAT PTPRM NDRG4 GCNT3 MUC13 EPAS1 HACD4 CYBRD1 PHLDB2 C2orf40 HSD17B6 GJA5 MYADM RPL39L SPINK1 PLAU S100P SLC6A4 TMEM204 HMGB3 DPYSL2 GOLM1 PROM2 GIMAP8 CDH13 GJA1 BTNL9 VEPH1 SAXO2 AADAC DACH1 ADAMDEC1 PTPRB PPARG MT1M TNNC1 CPB2 EMP1 SERINC2 CKS1B SLC19A3 AOC3 DES MS4A8 HS6ST2 MYCT1 ETV4 STK32A MYH10 STX11 LGSN SLPI RGCC VWF CRIM1 GABARAPL1 VSIG4 CYYR1 CDHR3 PLEKHH2 SEMA3B SCEL PBK HEG1 ANGPT1 SEMA6A DUOX1 FERMT2 CD34 ARHGAP31 MFAP4 ASPM NCKAP5 CD24 FAP PRG4 PLEK2 RBP4 EPB41L2 ADAMTS1 SLC7A5 AK4 SORBS1 IL37 LRCH2 LIFR STXBP6 IL3RA LIMCH1 DENND3 SPDEF GJB2 CALCRL RECK PLA2G4F NEXN THBS2 CA2 METTL7A ITM2A FAM189A2 LGI3 FMO2 ACTG2 PLLP HHIP ADH1B FERMT1 HMMR NEDD9 SMPDL3B CAB39L CFB PCP4 FCN3 STAC MUC21 KIF20A SEMA5A CST2 COL10A1 RAMP2 VIPR1 PLAC8 ANKRD1 GPA33 KCNJ15 LGR4 TTK KCNN4 CLDN18 NCAPG SLCO4C1 CP OLFML1 ATP10B CYP24A1 COL6A6 FRMD4B MMP9 |
