# Supplementary figures and images for: NCAPG promotes the progression of lung adenocarcinoma via the TGF-β signaling pathway
Source: Cancer Cell Int. 2021 Aug 21;21:443. doi: 10.1186/s12935-021-02138-w (PMC8380402; doi:10.1186/s12935-021-02138-w)

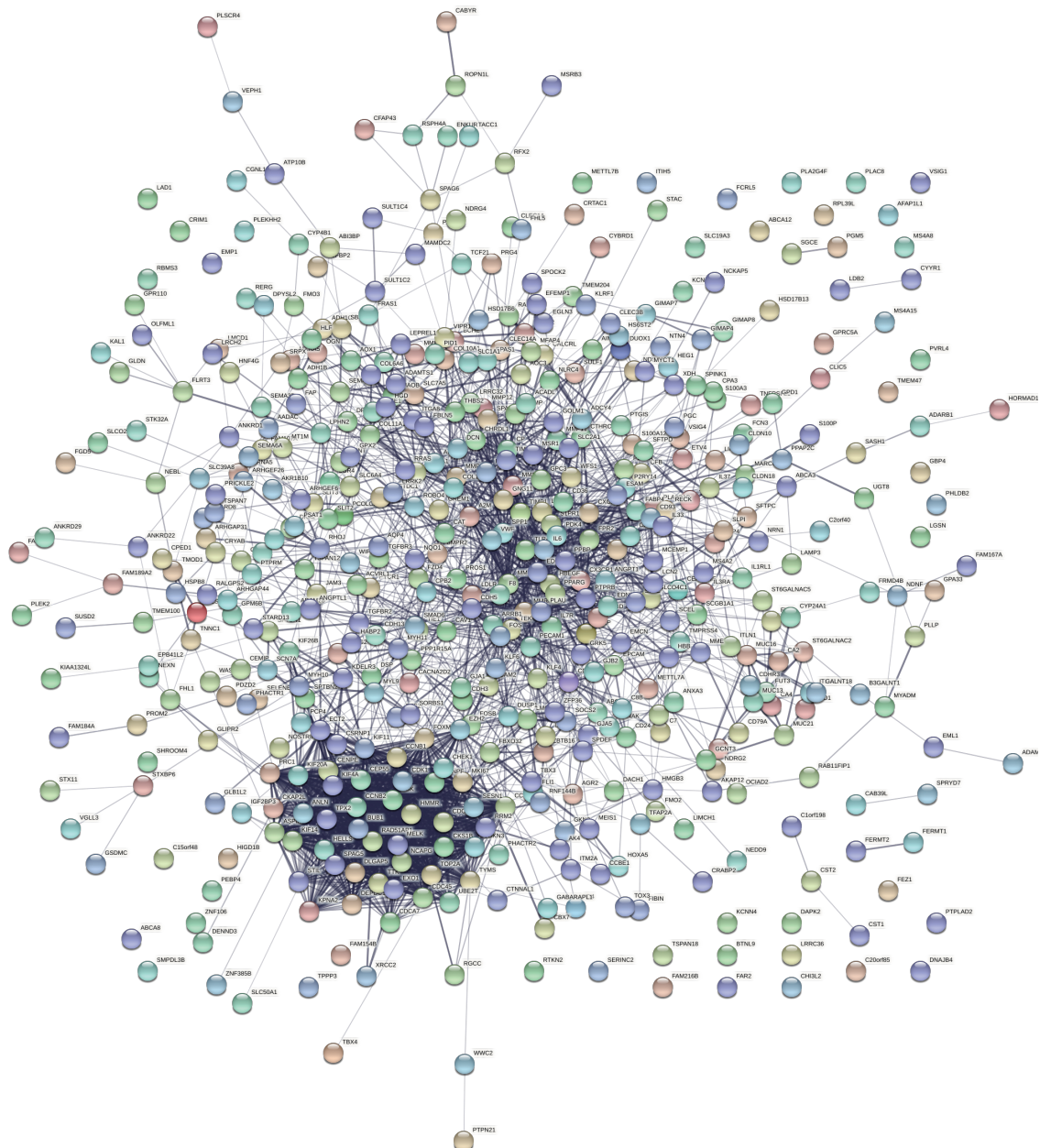

Supplement: Supplementary file 3 — Additional file 3: Figure S1. PPI network of overlapping genes. [file 12935_2021_2138_MOESM3_ESM.pdf]

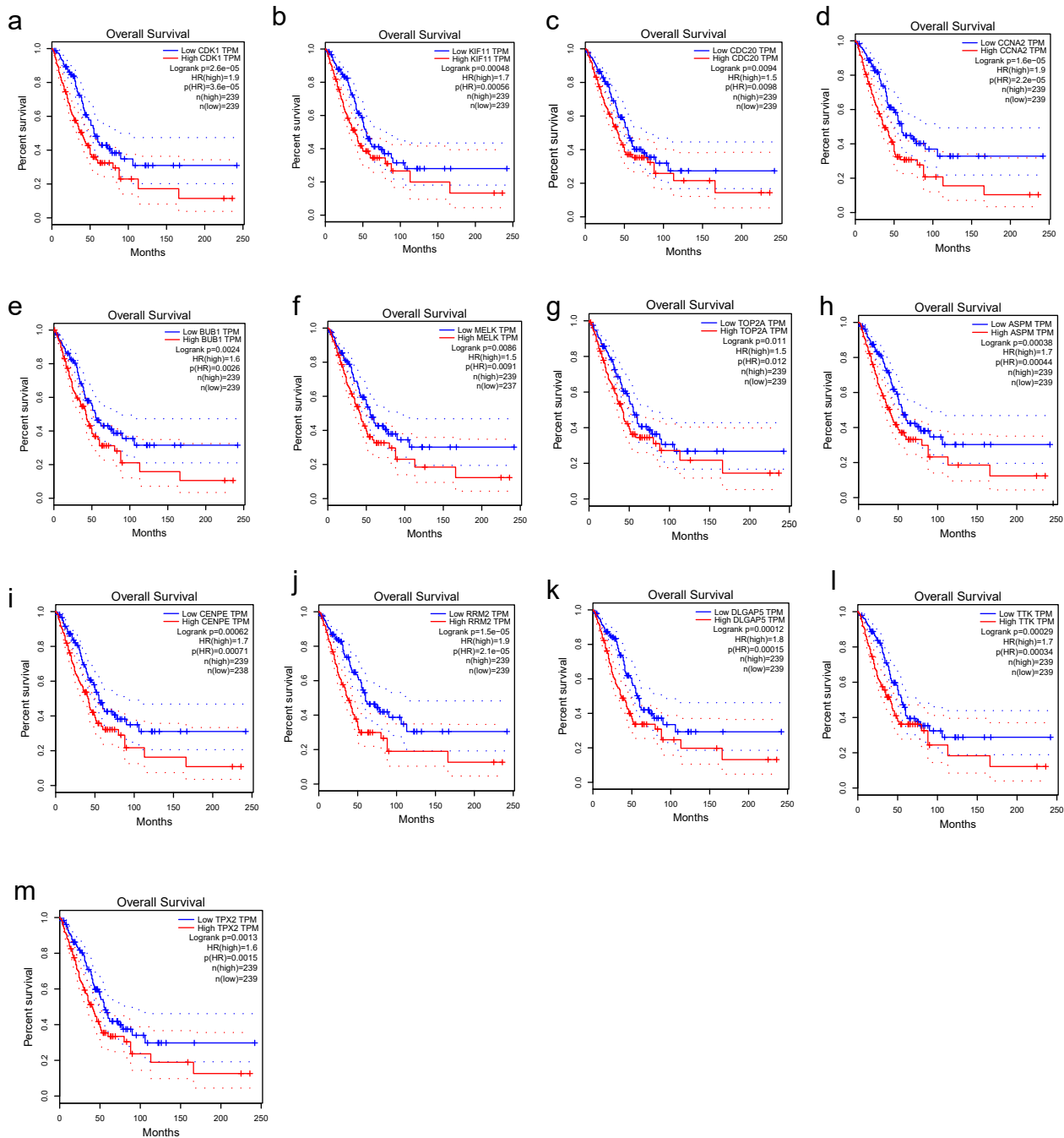

Supplement: Supplementary file 4 — Additional file 4: Figure S2. Kaplan–Meier overall survival analysis of the top 14 genes in patients with LUAD. [file 12935_2021_2138_MOESM4_ESM.pdf]
